# Collaborative Multi-Sensor Fusion for Intelligent Flow Regulation and State Monitoring in Digital Plunger Pumps

**DOI:** 10.3390/s26030919

**Published:** 2026-01-31

**Authors:** Fang Yang, Zisheng Lian, Zhandong Zhang, Runze Li, Mingqi Jiang, Wentao Xi

**Affiliations:** 1 Shanxi Key Laboratory of Fully Mechanized Coal Mining Equipment, College of Mechanical Engineering, Taiyuan University of Technology, Taiyuan 030024, China; yangfang0031@link.tyut.edu.cn (F.Y.);; 2 School of Mechanical and Electrical Engineering, Shanxi Datong University, Datong 037003, China

**Keywords:** digital plunger pump, multi-sensor collaboration, coupled model, flow control, intelligent fluid supply

## Abstract

To address the technical challenge where traditional high-pressure, large-flow emulsion pump stations cannot adapt to the drastic flow rate changes in hydraulic supports due to the fixed displacement of their quantitative pumps—leading to frequent system unloading, severe impacts, and damage—this study proposes an intelligent flow control method based on the digital flow distribution principle for actively perceiving and matching support demands. Building on this method, a compact, electro-hydraulically separated prototype with stepless flow regulation was developed. The system integrates high-speed switching solenoid valves, a piston push rod, a plunger pump, sensors, and a controller. By monitoring piston position in real time, the controller employs an optimized combined regulation strategy that integrates adjustable duty cycles across single, dual, and multiple cycles. This dynamically adjusts the switching timing of the pilot solenoid valve, thereby precisely controlling the closure of the inlet valve. As a result, part of the fluid can return to the suction line during the compression phase, fundamentally achieving accurate and smooth matching between the pump output flow and support demand, while significantly reducing system fluctuations and impacts. This research adopts a combined approach of co-simulation and experimental validation to deeply investigate the dynamic coupling relationship between the piston’s extreme position and delayed valve closure. It further establishes a comprehensive dynamic coupling model covering the response of the pilot valve, actuator motion, and backflow control characteristics. By analyzing key parameters such as reset spring stiffness, piston cylinder diameter, and actuator load, the system reliability is optimized. Evaluation of the backflow strategy and delay phase verifies the effectiveness of the multi-mode composite regulation strategy based on digital displacement pump technology, which extends the effective flow range of the pump to 20–100% of its rated flow. Experimental results show that the system achieves a flow regulation range of 83% under load and 57% without load, with energy efficiency improved by 15–20% due to a significant reduction in overflow losses. Compared with traditional unloading methods, this approach demonstrates markedly higher control precision and stability, with substantial reductions in both flow root mean square error (53.4 L/min vs. 357.2 L/min) and fluctuation amplitude (±3.5 L/min vs. ±12.8 L/min). The system can intelligently respond to support conditions, providing high pressure with small flow during the lowering stage and low pressure with large flow during the lifting stage, effectively achieving on-demand and precise supply of dynamic flow and pressure. The proposed “demand feedforward–flow coordination” control architecture, the innovative electro-hydraulically separated structure, and the multi-cycle optimized regulation strategy collectively provide a practical and feasible solution for upgrading the fluid supply system in fully mechanized mining faces toward fast response, high energy efficiency, and intelligent operation.

## 1. Introduction

The multi-plunger valved reciprocating positive displacement pump, distinguished by its high-pressure output, substantial flow capacity, and superior self-priming performance, has been widely deployed across mining machinery, marine engineering, and the petrochemical industry. In contemporary coal mining operations, this pump serves as a critical component within emulsion pump systems, supplying hydraulic power to drive support units through four principal operational cycles—namely, “lowering, shifting, raising, and advancing”—in addition to auxiliary functions. A significant technical challenge arises from the order-of-magnitude variations in fluid demand across different operational phases of hydraulic supports, necessitating the development of a supply system capable of wide-range flow regulation with millisecond-level responsiveness [1,2]. Conventional emulsion pumps, however, are inherently limited by their fixed-displacement design and non-adjustable geometric displacement—a constraint rooted in the fundamental working principles and structural configurations of such pumps. To mitigate this limitation, variable-frequency drive (VFD) technology, electromagnetic unloading systems, or hybrid methodologies combining both approaches are predominantly employed in current engineering practice to achieve precise flow regulation at the pump outlet [3].

Wang G et al. [4] developed an intelligent integrated fluid supply system for extra-large mining height working faces. Industrial validation at the Shendong Daliuta Coal Mine demonstrated a 15% improvement in comprehensive working face efficiency. Notwithstanding, the electromagnetic unloading–intelligent frequency conversion hybrid control strategy employed exhibits notable technical limitations: solenoid valves are prone to hydraulic shock under high-frequency switching conditions (>20 cycles/min), with peak pressure fluctuations reaching ±2.5 MPa, while the frequency conversion motor drive system exhibits prolonged dynamic response times—up to 300 ms—due to excessive rotational inertia (J = 0.85 kg·m^2^), thereby impeding the fulfillment of transient flow regulation requirements [5,6].

Dong J et al. [7] systematically reviewed the technological evolution of hydraulic valves, highlighting the achievement of sub-5 ms response times in high-speed switching electromechanical actuators through innovations such as novel soft-magnetic materials and multilayer laminated structures. However, stand-alone high-speed switching valves are constrained by single-orifice designs, typically limited to rated pressures below 35 MPa and flow capacities under 30 L/min, thereby precluding direct integration with high-pressure, large-flow emulsion pump systems [8,9]. Chapple, P. et al. [10] conducted a systematic analysis of topological innovations in digital valves, reporting a 40% improvement in magnetic flux density via Halbach array permanent magnet designs in electromagnetic aspects, and a 28% reduction in pressure loss through three-stage throttling groove optimization in valve body design. Despite these advances, current research remains predominantly focused on component-level valve optimization, without achieving deep integration of electromechanical actuators within emulsion pump architectures, leading to approximately 12% additional system energy loss [11,12]. Wang, T. et al. [13] proposed a digital transformation of suction valves using electromechanical actuators, converting conventional fixed-displacement positive displacement pumps into adjustable systems via electromechanical–hydraulic coupling. Numerical simulations indicate that this design attains system response speeds on the order of 50 ms; however, experimental validation of durability and pressure pulsation suppression under real operating conditions remains lacking [14,15,16]. Dindorf, R et al. [17] pioneered an innovative parallel suction valve topology to enable dynamic flow regulation in plunger chambers via pulse-width modulation control. Experimental results indicate that this approach achieves 90% setpoint tracking within 200 ms under step flow demand conditions. However, the theoretical model does not adequately elucidate the multi-valve cooperative flow distribution mechanism, nor does it account for the influence of load pressure fluctuations (Δp = ±15% of rated pressure) on regulation accuracy, resulting in steady-state flow deviations of up to ±8% [18,19].

The stepless capacity control method is applied to piston compressors. This method utilizes hydraulically actuated unloaders to delay the closing of the suction valves, allowing a portion of the gas to return, thereby modifying the output capacity. Compared to constant-capacity operation, capacity regulation significantly affects the dynamic characteristics of the valves and the thermodynamic cycle of the compressor. In this context, Yu et al. [20] treated the valve as a hydrodynamic component, establishing a pump-valve coupling equation. They further derived the differential equation of motion for reciprocating pump valves by integrating the kinematics of the plunger and the valve. Based on different operating conditions, they developed practical simulation models and systematically analyzed the output flow and pressure fluctuation characteristics. Lu et al. [21] constructed a model of the regulation device coupled with the intake valve motion, focusing on the coupling relationship between the plunger displacement and the opening/closing characteristics of the distribution valve. Their work revealed the hysteresis in the actuation of pump valves. However, the aforementioned studies primarily concentrate on the influence of structural parameters and on steady-state or simple transient conditions with small flow rates. For digital hydraulic systems, where the coordinated opening and closing of multiple high-speed on/off valves generates extremely complex and rapid flow transients, the coupled mechanisms underlying system-level pressure shocks and component damage, along with effective control strategies, remain key research challenges.

Based on the aforementioned research background and status, this study aims to address the key issue of traditional fluid supply systems struggling to actively match the drastic flow rate changes in hydraulic supports. By constructing an intelligent “coarse-fine coordination” control strategy, it achieves precise flow regulation while significantly reducing component actuation frequency, thereby effectively suppressing hydraulic shocks and cavitation damage, and enhancing system reliability. To achieve these objectives, this study systematically carried out the following work: Firstly, the mechanism of dynamic flow regulation in emulsion pumps was clarified through numerical simulation and experiments. Building on this, a novel digital flow control method and its implementation framework based on electro-hydraulic cooperative control were proposed. Adopting an integrated electromechanical-hydraulic design philosophy, a co-simulation platform for digital flow regulation of emulsion pumps, incorporating a high-speed switching valve actuator, was constructed. This actuator directly regulates the dynamic behavior of the suction valve, enabling multi-objective optimization design and extending valve lifespan. Addressing the inherent multi-domain coupling characteristics (mechanical-hydraulic-electrical) of the system, for which traditional single-domain modeling methods struggle to accurately describe parameter coupling effects, this paper innovatively employed interface-based multiphysics co-simulation technology. A holistic model of the digital valve system encompassing electromechanical-hydraulic coupling effects was established. The study focused on investigating the pump-valve interaction mechanisms under different operating conditions and optimized key parameters, thereby verifying the feasibility and effectiveness of the proposed control scheme. The contribution of this research lies in providing a clear technical pathway for achieving precise and flexible regulation at the flow source in hydraulic systems. It offers theoretical support and design principles at both the methodological and systemic levels for fundamentally solving the long-standing challenges of “supply-demand mismatch” and “high-frequency shocks.” This holds significant theoretical value and engineering importance for advancing hydraulic technology towards precision, high efficiency, and high reliability.

## 2. Theoretical Model Construction

### 2.1. Research Framework

Digital distribution technology represents not only a pivotal solution to the high energy consumption and low efficiency prevalent in current emulsion pump stations, but also constitutes the fundamental basis and inevitable pathway for developing the next generation of intelligent, efficient, and environmentally sustainable fluid supply systems. Conventional emulsion pump stations utilize constant-speed motor-driven fixed-displacement pumps, which are inherently constrained by the limitation of “non-adjustable displacement.” Their dependence on bypass overflow or unloading valves for pressure and flow regulation leads to an operation mode defined by “oversupply relative to demand” and “compulsory overflow,” resulting in substantial energy losses and sluggish pressure-stabilization response. Such a configuration is inadequate to address the complex and transient fluid requirements of hydraulic support systems.

By incorporating electromagnetic actuation mechanisms and advanced control strategies, along with operational data acquired from a digital pump monitoring and control system and structural parameters of conventional pumps, multiple representative fluid demand scenarios were simulated. Through precise modulation of the de-energization timing of pilot-operated high-flow high-speed switching valves, accurate regulation of the displacement of traditional emulsion pumps has been realized.

**Figure 1:**
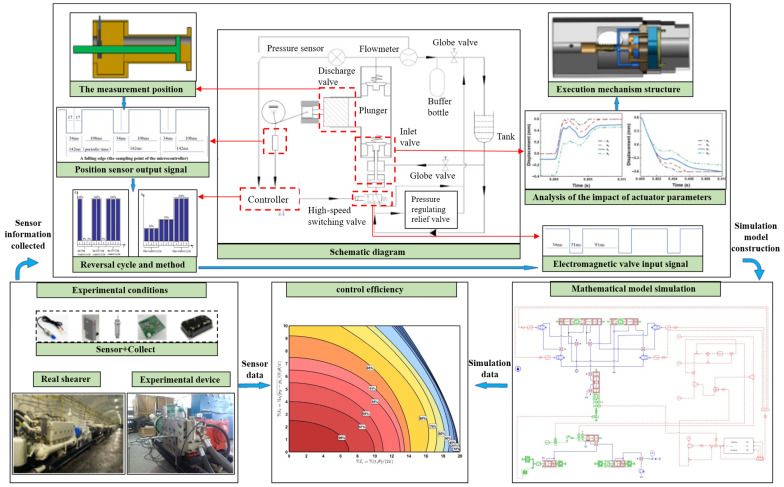
Research framework of the digital pump measurement and control system.

This achievement offers crucial theoretical foundations and practical insights for the design and optimization of intelligent digital fluid supply systems. As illustrated in Figure 1, the overall framework is outlined as follows:

(1) The principal functions and technical specifications of the digital distribution system were analyzed and computed. A high-flow high-speed switching valve and its associated controller were deployed to achieve digital regulation of the effective displacement of the plunger pump, culminating in the conceptual design of the system and thereby enabling variable displacement control of the fixed-displacement pump. Plunger position is detected and ascertained via a Hall effect sensor. When the plunger of the single-cylinder pump initiates its descent from top dead center, the controller energizes the pilot solenoid valve. High-pressure fluid from the pilot supply pump traverses the pilot valve into the piston chamber of the high-flow solenoid valve, actuating the push rod to open the plunger pump’s inlet valve, thereby permitting fluid intake into the plunger chamber. As the plunger ascends from bottom dead center, the pilot-operated high-flow switching valve remains open, and the fluid pressurized by the plunger recirculates to the inlet side via the inlet valve. When fluid delivery to the high-pressure chamber is required, the controller de-energizes the pilot valve. The inlet valve closes, and the outlet valve opens correspondingly, enabling fluid discharge into the high-pressure pipeline. Throughout the plunger’s compression stroke, precise displacement control is accomplished by adjusting the de-energization timing of the pilot-operated high-flow high-speed switching valve. Once the main valve piston chamber is replenished with low-pressure fluid, the piston resets to bottom dead center under spring force. At this stage, the pilot solenoid valve deactivates, and the pump reverts to operation based on mechanical distribution principles.
(2) Focusing on the newly integrated actuator within the digital distribution system, this research entailed structural design and parametric analysis, and proposed a multi-mode composite reflux regulation strategy in accordance with actual fluid usage conditions and operational characteristics. During the actuator’s structural design phase, the influence of critical parameters—such as piston diameter and spring stiffness—on its dynamic behavior was ascertained. Subsequently, a systematic investigation was conducted into the mechanism by which dynamic parameters, including opening force and displacement, affect the response characteristics of the suction valve during opening and closing sequences. Ultimately, grounded in multi-objective optimization theory, a composite regulation strategy was formulated, integrating single-cycle uniform loading, dual-cycle differential loading, multi-cycle full-plunger operation, and adjustable duty cycle control. Focusing on the newly introduced actuator in the digital distribution system, this study carried out structural design and parameter analysis, and proposed a multi-mode composite reflux regulation strategy in combination with actual fluid usage conditions and operational characteristics. During the structural design phase of the actuator, the influence of key parameters such as piston diameter and spring stiffness on its motion behavior was clarified. Subsequently, a systematic analysis was conducted on the mechanism through which dynamic parameters—such as the opening force and displacement—affect the response characteristics of the suction valve during opening and closing. Finally, based on multi-objective optimization theory, a composite regulation strategy was developed that integrates single-cycle uniform load, dual-cycle differential load, multi-cycle full-plunger operation, and adjustable duty cycle control.
(3) Within an experimental setup, this study established a digital pump measurement and control system utilizing a conventional plunger pump and a newly designed actuator prototype as the physical entity. The system enables real-time monitoring of parameters such as plunger displacement, discharge pressure, and flow rate, effectively mitigating the challenges of non-adjustable displacement in fixed-displacement pumps and energy wastage due to relief valve overflow. By comparing the displacement regulation performance of the digital pump prototype under varying operational conditions and examining the interrelationship among digital valve response time, fluid action area, and system efficiency, the feasibility of the proposed digital distribution methodology was empirically validated.

### 2.2. Mathematical Model of the Suction Valve

**Figure 2:**
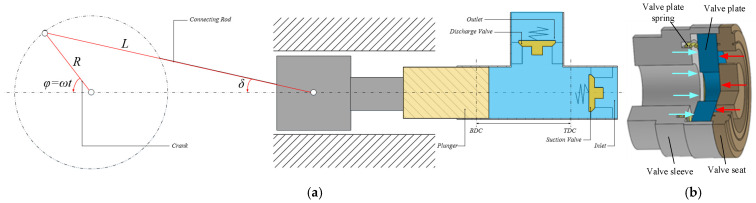
Geometric relations and motion models: (**a**) Single-plunger chamber; (**b**) Suction valve.

Figure 2a illustrates the flow channel configuration within a single-plunger chamber of the emulsion pump. The opening and closing behavior of the distribution valve is governed by the reciprocating motion of the plunger, with periodic pressure fluctuations inside the plunger chamber acting as the primary driver for valve spool displacement. The coordinate system is defined with the origin at the minimum stroke position of the plunger, and the positive direction aligned with plunger motion that increases the chamber volume. Kinematic analysis of both the plunger and the distribution valve facilitates the derivation of a flow continuity equation specific to the individual plunger chamber. This formulation incorporates pressure and flow variations resulting from both plunger motion and valve spool displacement. Furthermore, the dynamic response of the distribution valve is characterized by coupling the force equilibrium equation of the spool with the flow equation through the valve orifice.

The instantaneous pressure inside a single-plunger chamber is determined using the integral form of the continuity equation [22,23]:

(1) $$\frac{dP_{vol}}{dt}=(Q_{s}-Q_{p}-Q_{d})\frac{E}{V_{vol}}$$

In the equation: *P_vol_* denotes the instantaneous pressure in the plunger chamber, *E* represents the bulk modulus of the fluid, *Q_s_* and *Q_d_* correspond to the instantaneous volumetric flow rates through the suction and discharge valves, respectively, and *Q_P_* refers to the rate of volume change due to plunger motion. The instantaneous chamber volume *V_vol_* can be expressed as [24,25]:

(2) $$Q_{p}=v_{p}A_{p}$$

(3) $$V_{vol}=A_{p}x_{p}+V_{d}$$

where *A_P_* is the cross-sectional area of the plunger, and *V_d_* is the dead volume of the plunger chamber.

The flow continuity equation across the valve orifice is given by [26]

(4) $$Q_{0}=[C_{d}\pi dx\sqrt{2\left|P_{1}-P_{2}\right|/\rho }]sign\left(P_{1}-P_{2}\right)$$

Accounting for the Westphal phenomenon, the instantaneous flow rate through the valve orifice can be formulated as [26]

(5) $$Q_{i}=[C_{d}\pi dx\sqrt{2\left|P_{1}-P_{2}\right|/\rho }]sign(P_{1}-P_{2})+\frac{dx}{dt}A_{V}$$

Here, *C_d_* is the discharge coefficient, *d* is the orifice diameter, *x* is the spool displacement, and *Av* is the effective flow area of the spool. The sign function determines the flow direction: $sign=\left\{\begin{array}{c} 1;P_{1}>P_{2}\\ -1;P_{1}<P_{2} \end{array}\right.$; if *P*_1_ > *P*_2_, the function returns +1, indicating forward flow; otherwise, a negative value signifies reverse flow.

### 2.3. Mathematical Model of the Unloader

**Figure 3:**
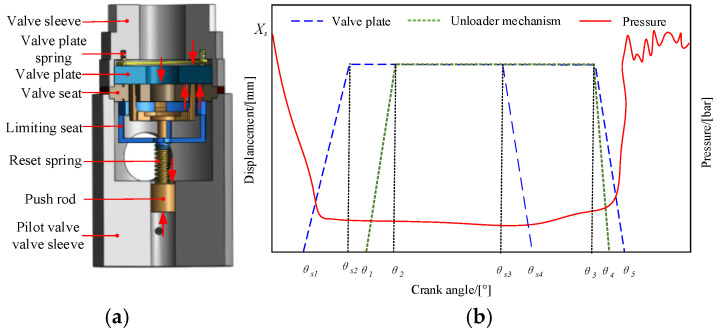
Structural relationship diagram: (**a**) Force analysis, (**b**) Relationship diagram of suction valve displacement, unloader displacement, and plunger chamber pressure.

As illustrated in Figure 3, the unloading mechanism is composed of the following components arranged sequentially from bottom to top: pilot valve sleeve, push rod, reset spring (whose parameters are provided in Table 1; note that the spring acts as a resistance source during push rod extension and as a driving source during retraction), limit seat, valve seat, inlet valve plate, valve plate spring, and valve sleeve. The operational principle can be summarized as follows: upon signal reception, the solenoid valve activates, driving the push rod upward, which subsequently lifts the inlet valve plate and permits fluid intake into the plunger cavity. As the plunger ascends from the bottom dead center (BDC), the solenoid valve remains energized, thereby maintaining the inlet valve plate in the open position. As a result, the fluid displaced by the plunger returns via the inlet valve to the inlet port. When high-pressure fluid delivery is required, the solenoid valve is de-energized, leading to the closure of the inlet valve plate and the concurrent opening of the outlet valve, thereby directing the fluid into the high-pressure line.

As the primary actuating element that applies forced mechanical action, the kinematic behavior of the unloader is intrinsically linked to the motion of the valve plate. Therefore, formulating the motion equation of the unloader is essential for analyzing the influence of mechanical system characteristics on the overall performance of the digital flow control system [27,28,29].

The force analysis of the unloader is depicted in Figure 3a. During the ejection phase, the unloader is subjected to hydraulic force, spring force, frictional force, among others. The governing differential equations for the ejection and retraction phases of the unloader are given as follows:

(6) $$M_{\mathrm{mu}}\frac{d^{2}x_{\mathrm{mu}}}{dt^{2}}=\left\{\begin{array}{l} P_{h}-f-F_{\mathrm{smu}}\;\;t_{1}\leq t\leq t_{2}\\ 0\;\;\;\;\;\;\;\;t_{2}\leq t\leq t_{3}\\ F_{\mathrm{smu}}+F_{L}-f\;\;t_{3}\leq t\leq t_{4} \end{array}\right.$$

where *t*_1_ represents the initiation time of unloader ejection, *t*_2_ denotes the completion time of unloader ejection, *t*_3_ indicates the initiation time of unloader retraction, *t*_4_ signifies the completion time of unloader retraction, *M_mu_* is the mass of the unloader’s moving components, *x_mu_* represents the unloader displacement, *P_h_* stands for the hydraulic driving force, *f* denotes the total friction force, *F_smu_* indicates the spring force, *F_L_* is the ejection force from the hydraulic cylinder.

Figure 3b illustrates the displacement relationship between the unloader of the flow regulation system and the valve plate. During normal operation of the emulsion pump, the plunger, driven by the crankshaft, initiates a return stroke. This action increases the volume of the working chamber and reduces the pressure within the plunger cavity. Once the pressure drops below atmospheric level, the plunger cavity enters a negative pressure state. When the cavity pressure decreases to a critical threshold where the net hydraulic force exceeds the resistance provided by the suction valve spring, the suction valve plate begins to open ($\theta_{s1}∼\theta_{s2}$), marking the commencement of the pump’s suction process. As the plunger undergoes another reversal in motion, the emulsion within the plunger cavity is compressed, leading to a rise in internal pressure. Once the pressure increases sufficiently such that the net hydraulic force acting on the suction valve plate can no longer overcome the spring force, the suction valve plate closes ($\theta_{s3}∼\theta_{s4}$).

When the emulsion pump is integrated with a continuous variable displacement regulation system, the flow regulation system must complete the ejection action prior to the start of the discharge stroke ($\theta_{1}∼\theta_{2}$). The timing control is governed by the dynamic response characteristics of the flow regulation system. During the discharge stroke, through active adjustment by the flow regulation system, the suction valve plate remains fully open, allowing a portion of the emulsion to flow back through the suction valve to the suction pipeline inlet. Once the flow rate meets the preset requirement, the flow regulation system activates accordingly ($\theta_{3}∼\theta_{4}$), and the external driving force applied to the suction valve plate is released. The suction valve plate then returns to its initial closed state ($\theta_{3}∼\theta_{5}$), thereby achieving flow regulation at the outlet of the emulsion pump. The duration of the backflow phase ($\theta_{s3}∼\theta_{5}$) determines the operational workload under flow regulation conditions.

## 3. Joint Simulation Process

This co-simulation model is designed to address the critical challenge of traditional hydraulic fluid supply systems in actively adapting to drastic flow variations required by hydraulic supports. By establishing an Amesim-Simulink (2021.1) collaborative simulation platform, it aims to validate an intelligent control strategy based on the digital flow distribution principle. Within this framework, Amesim is responsible for modeling the core hydraulic components (such as the plunger pump, high-speed switching valves, and suction valves), accurately characterizing fluid dynamic behavior and its coupling with mechanical motion. It outputs key physical state variables—like piston displacement and chamber pressure—to Simulink via an interface module. Simulink, in turn, implements a multi-cycle duty cycle adjustment algorithm to generate precise switching timing signals for the pilot solenoid valves and incorporates physical state feedback from Amesim to achieve closed-loop control. Through this electromechanical-hydraulic multi-domain co-simulation approach, the model enables dynamic analysis of the coordinated pump-valve regulation process. Ultimately, it evaluates performance metrics including flow control accuracy, system shock suppression capability, and energy efficiency optimization, thereby providing theoretical support for the intelligent upgrading of hydraulic systems.

The physical model of the digital flow control system is depicted in the accompanying schematic diagram. Although researchers frequently employ simplified simulation models of individual subsystems to analyze parameter variations, this conventional methodology exhibits significant limitations [30,31]. Crucially, inherent coupling effects between subsystems—arising from parameter interdependencies—establish a complex dynamic in which adjustments to one subsystem’s parameters not only influence its own performance but also induce characteristic alterations in interconnected systems. Such cross-system interactions often result in progressive performance degradation of the overall system.

**Figure 4:**
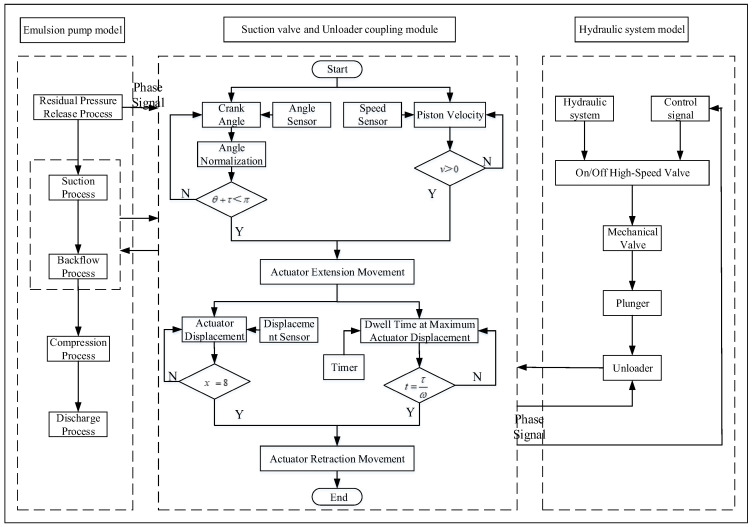
Flowchart of co-simulation scheme for digital flow control system.

In this study, the critical technical challenge of performing coupling analysis among multiple subsystems in flow control systems is addressed through the development of a co-simulation scheme based on interface-driven multi-software collaborative simulation technology, as illustrated in Figure 4. The joint simulation process necessitates the resolution of two key issues: ensuring synchronization across software platforms and determining the interactive parameters between subsystems [32,33]. To overcome these challenges, the following strategies are implemented: (1) The phase output corresponding to the piston motion stop point in the pump model is adopted as the clock synchronization reference for co-simulation, with fixed-step integration employed to maintain inter-software synchronization. (2) Through comprehensive analysis of the operational principles governing the digital flow control system, critical interface parameters—including interaction forces, displacements, and velocities—that characterize inter-model interactions are identified, while high sampling frequency is implemented to ensure superior simulation accuracy. For instance, in an emulsion pump with a spindle speed of 422 r/min, the reciprocating motion cycle T of the piston is 142 ms, and the compression stroke occupies approximately half of this cycle (71 ms). From a dynamic control standpoint, to ensure timely opening and closing of the suction valve for continuous discharge flow regulation, the actuator’s opening response delay must be constrained within 25 ms, corresponding to a minimum sampling frequency of 40 Hz.

**Figure 5:**
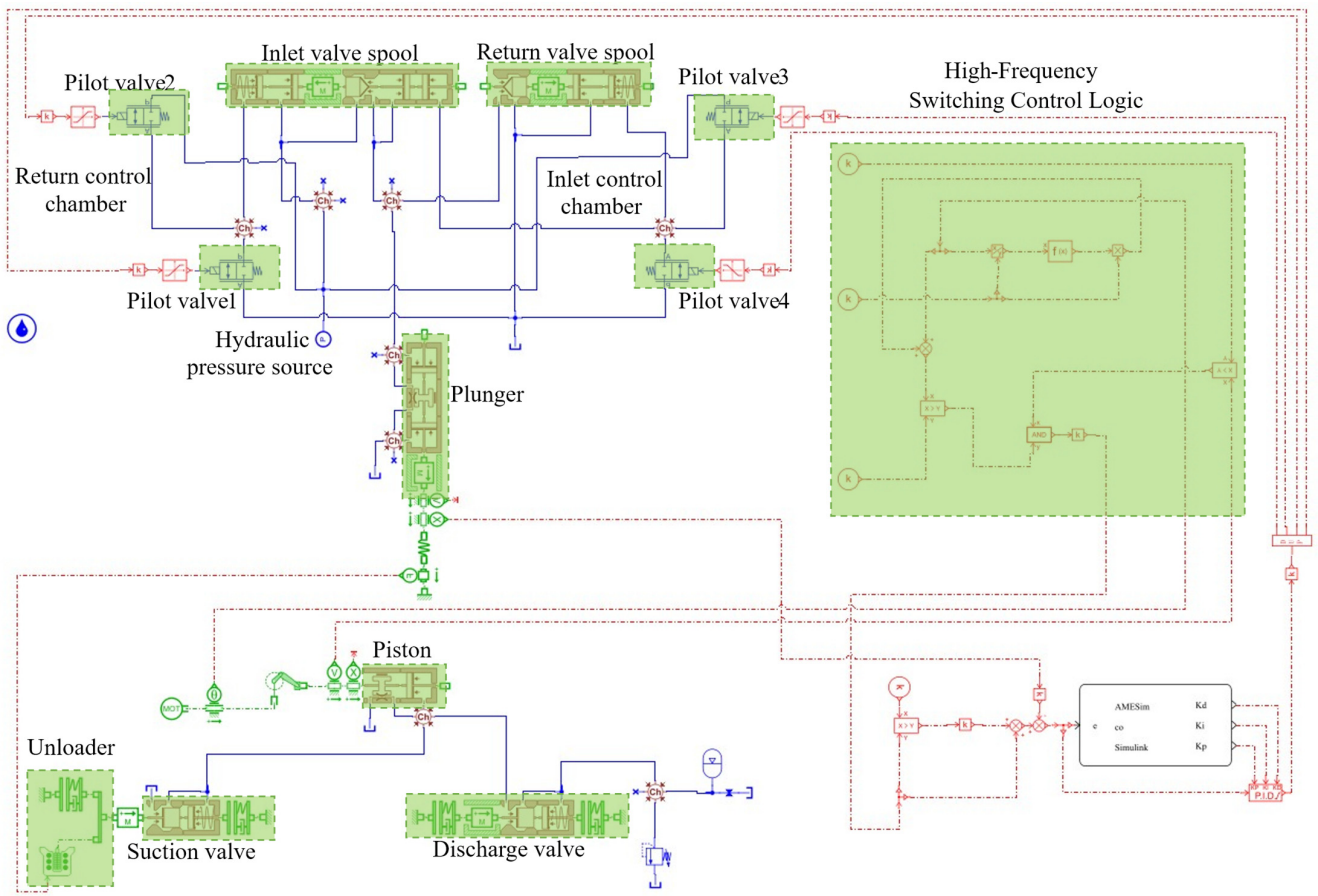
Closed-loop simulation model for high-frequency switching control.

Within the co-simulation modeling framework, the on/off timing of the solenoid valves is primarily determined by the duty cycle modulation of control signals, which directly governs the duration of hydraulic force application on the actuator and consequently induces significant modifications in its dynamic response characteristics. The architecture of the closed-loop simulation model for high-frequency switching control is presented in Figure 5. A three-dimensional interface parameter system—comprising interaction force, displacement, and velocity—has been established to accurately characterize the coupling mechanisms among subsystems. The actuator control efficacy evaluation system, developed through mathematical modeling, enables precise determination of the spool control status via synergistic analysis of three key parameters: the relative position between the unloader and the spool, the relative velocity of the actuator, and the actuation duration [34,35,36]. When the actuator assumes control dominance, active modulation of the spool’s movement trajectory directly influences the dynamic performance metrics of the digital control system. This mechano-hydraulic coupling effect can be quantitatively characterized using the established parametric evaluation system.

During actual operation of the emulsion pump, the closing timing of the suction valve spool is actively regulated during the plunger discharge stroke via a valve-controlled cylinder actuator, in accordance with the digital flow distribution principle de-scribed earlier. This delayed closing mechanism enables the plunger to redirect the emulsion back to the inlet manifold. The underlying operational principle is as follows: during the actively delayed phase of suction valve closure, the pressure gradient developed within the plunger chamber remains insufficient to overcome the discharge valve’s opening threshold, resulting in a zero-flow output state throughout this interval.

By systematically implementing sequential delayed closing control for the suction valves across all plunger chambers of the emulsion pump, precise regulation of the total output flow can be achieved. The dynamic processes associated with the three working cycle modes of a single-plunger chamber are described as follows:

### 3.1. Single-Plunger Operating Cycle Modes

(1) Normal Operating Cycle

**Figure 6:**
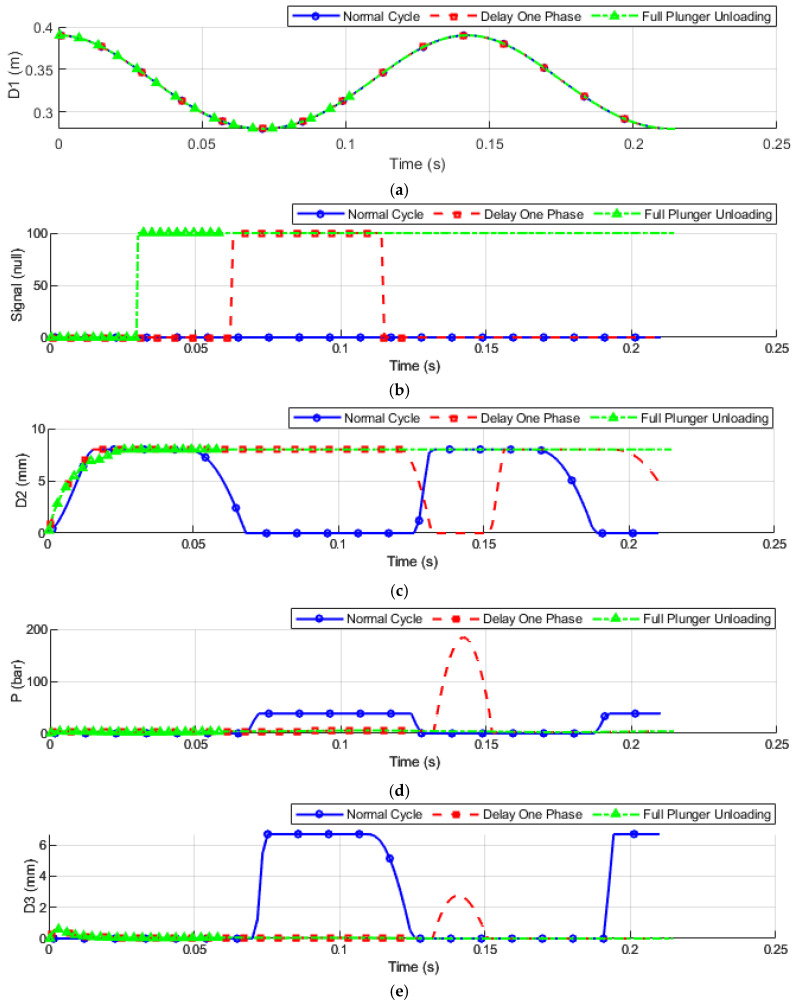
Comparison of characteristics under different unloading modes: (**a**) Plunger displacement, (**b**) Solenoid valve signal, (**c**) Inlet valve displacement, (**d**) Intake chamber pressure, (**e**) Exhaust valve displacement.

The experimental results presented in Figure 6 demonstrate that under this operational mode, the actuator remains entirely inactive, while the suction valve performs standard opening and closing actions under the coupled influence of the upstream/downstream pressure differential, spring preload force, and the self-weight of the spool. The specific behavioral characteristics are summarized as follows: (a) Throughout the entire discharge stroke, the pressure within the plunger chamber remains consistently elevated, dictated by the external load; (b) The plunger continuously performs work on the emulsion across the full discharge stroke; (c) The flow rate discharged through the outlet valve strictly adheres to the rated capacity, as determined by the geometric dimensions and kinematic parameters of the plunger chamber.

(2) Intra-Plunger Phase Delay

Figure 6 reveals the following operational characteristics: the actuator initiates its intervention no later than the point at which the suction valve spool begins closing from its maximum opening position. During this phase, the valve-controlled cylinder completes its extension motion and resets within the remaining portion of the cycle. Throughout this actively delayed closure period, the suction valve is held open by the mechanical support provided by the actuator, and only closes normally once the cylinder retracts and support is withdrawn. Correspondingly, the emulsion is compressed to high pressure levels by the plunger only after the suction valve has fully closed. During the delayed closure interval, the chamber pressure remains relatively low—sufficient only to overcome the return flow resistance to the fluid reservoir.

Within a single-plunger chamber’s partial working cycle, the discharged flow rate correlates with the proportion of the delayed closure phase relative to the total discharge stroke duration.

(3) Single-Plunger Complete Unloading

Analysis of the experimental data in Figure 6 leads to the following conclusions: throughout the entire discharge stroke cycle, the suction valve remains persistently open due to full-cycle phase-delay control implemented by the actuator.

This operational mode exhibits three distinctive characteristics: (a) A critical low-pressure level insufficient to open the discharge valve is consistently maintained by the plunger chamber pressure; (b) Complete flow back of the emulsion medium to the fluid reservoir is achieved through the suction valve and inlet manifold passage; (c) Consistent zero output flow is maintained at the discharge valve. Through comparative analysis of the three working cycle modes, it is demonstrated that activation of the delayed-closure control strategy for the suction valve occurs exclusively during low-pressure conditions in the plunger chamber. Significant advantages for the simplification of actuator design are provided by this characteristic.

### 3.2. Multi-Plunger Operating Cycle Modes

Under actual operating conditions, multiple emulsion pumps are typically integrated to achieve cooperative fluid supply. Taking a five-plunger pump as an example, the system’s displacement per unit time is collectively determined by the number of pumps, the number of plungers, and the configuration of the cyclic sequence. Adjusting only one of these parameters—whether the number of pumps, plungers, or the cyclic sequence—enables only stepped regulation of displacement. In contrast, combined modulation of the displacement contribution from all three factors allows for continuous (stepless) regulation of the pump’s output. Based on this principle, this paper uses plunger count adjustment as an exemplar—noting that the regulation principles for pump number and cyclic sequence are analogous—and proposes three reflux regulation strategies: whole-plunger unloading, uniformly distributed reflux among plungers, and non-uniformly distributed reflux among plungers.

(1) Complete Unloading of Selected Plungers

**Figure 7:**
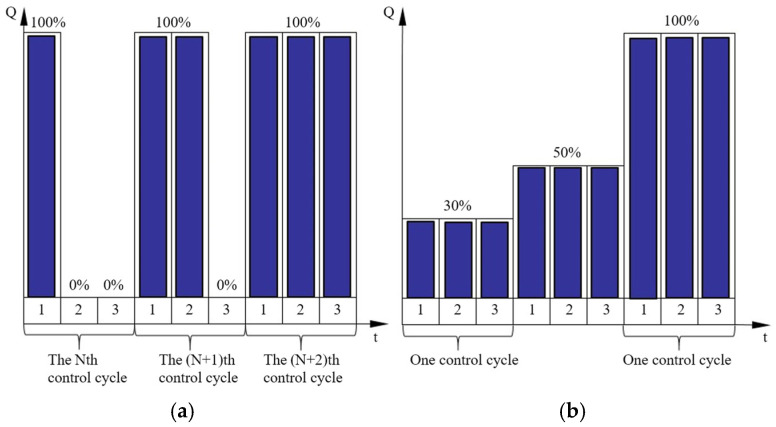
Reflux regulation methods: (**a**) Multi-cycle full-plunger recirculation regulation, (**b**) Single-cycle load-equalizing recirculation regulation.

As illustrated in Figure 7a, the multi-cycle full-plunger reflux regulation strategy employs a staged control procedure. Initially, complete unloading is applied to a selected plunger by maintaining its suction valve in a forced-open state via the actuator throughout the operating cycle, thereby establishing full-cycle reflux conditions. The system continuously monitors the discharge flow in a closed-loop manner: if the current reflux meets system requirements, the operating state is preserved; otherwise, the unloading function is sequentially activated for additional plungers in subsequent cycles until the target flow is achieved. Compared to single-cycle uniform load regulation, this approach offers significant engineering advantages in terms of dynamic response speed and control system reliability.

(2) Equal Distribution of Return Flow Among Plungers

The single-cycle uniform load reflux regulation method utilizes a closed-loop flow control strategy, characterized by its ability to precisely match the discharge volume per operating cycle with the real-time target flow value. As shown in Figure 7b, this mode enables highly uniform flow output from the plunger pump, manifested by: (a) minimal fluctuation in discharge pulse amplitude per unit time, (b) significant attenuation of system pressure pulsation, and (c) optimal flow regulation performance.

However, this precision control entails certain engineering trade-offs: the system must execute a composite action sequence—comprising actuator extension, steady-state maintenance, and rapid retraction—accurately within each operating cycle. This imposes stringent design requirements on both control system reliability and component service life.

(3) Unequal Distribution of Return Flow Among Plungers

**Figure 8:**
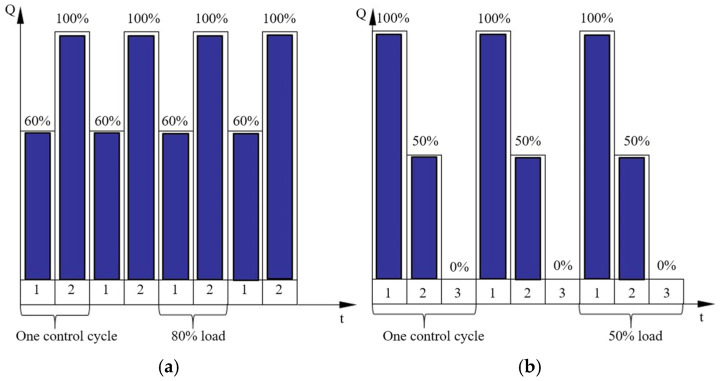
Reflux regulation methods: (**a**) Dual-cycle differential recirculation regulation, (**b**) Multi-cycle adjustable duty cycle differential recirculation regulation.

The dual-cycle differential load reflux regulation method adopts a modular flow control strategy based on two consecutive working cycles (0–720° crank angle), which together constitute a fundamental regulation unit. The total system flow results from the vector superposition of the flow contributions from each cycle within the unit. As depicted in Figure 8, this regulation mode incorporates three characteristic load states: (a) Full-load condition: the suction valve operates with standard opening and closing actions; (b) No-load condition: the actuator maintains the suction valve in a forced-open position throughout the cycle; (c) Partial-load condition: precise flow regulation is achieved by controlling the phase delay angle of the suction valve closure.

The system first calculates the required number of regulation units based on the target reflux flow, then dynamically optimizes the load ratio combination across these units using a load distribution algorithm. Within this framework: (a) Baseline flow is supplied by full-load cycles; (b) Maximum reflux is attained via no-load cycles; (c) Flow fine-tuning is achieved through partial-load cycles. Ultimately, precise flow control is realized by an optimized combination of these three operational states.

## 4. Experimental Platform Setup and Dynamic Analysis

### 4.1. Experimental Platform Setup

This study investigates the flow regulation characteristics of emulsion pumps under diversified working conditions, with concurrent validation of the effectiveness of the established numerical model.

To this end, a dedicated experimental platform for emulsion pump flow regulation systems was designed and constructed. Table 2 and Table 3 summarize the key configuration parameters of the experimental setup, including the geometric dimensions of the emulsifying pump, operating parameters, and the technical specifications of the sensors used.

**Figure 9:**
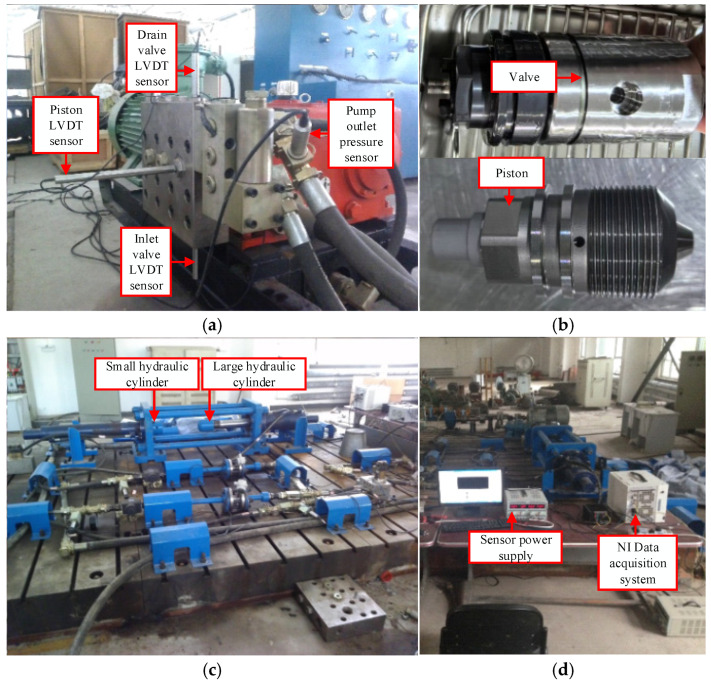
Emulsion pump sensor layout and data acquisition system: (**a**) Sensor position, (**b**) Actuator, (**c**) Load equipment, (**d**) Collection system.

Figure 9a illustrates the installation layout of pressure sensors and LVDT displacement sensors on the emulsion pump body. The newly integrated valves and pistons are displayed in Figure 9b. Load conditions are applied using both small and large hydraulic cylinders, as depicted in Figure 9c. A systematic arrangement of test equipment at the experimental site, including the NI data acquisition system and sensor power supply modules, is presented in Figure 9d.

**Figure 10:**
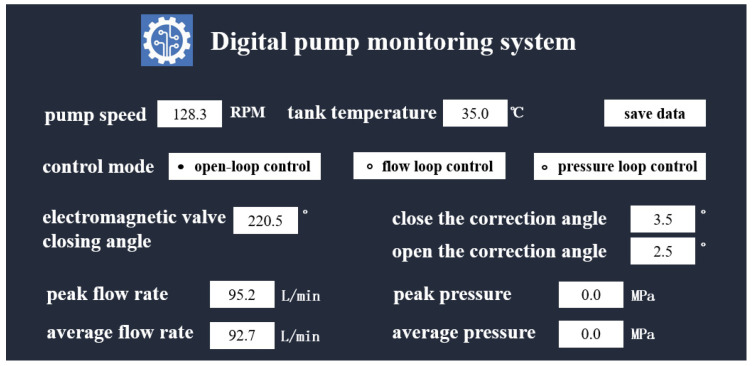
Digital pump monitoring system.

To examine the dynamic influence of the electromagnetic closing phase on the digital flow distribution system, a dedicated data acquisition system was developed, as shown in Figure 10. The core functionality of this system lies in achieving synchronous control and acquisition of multi-channel signals. The system controller receives three PWM input control signals and generates three corresponding drive outputs. Within each control cycle, a unified timebase ensures synchronous acquisition of multi-dimensional data—including critical parameters such as pump inlet/outlet pressure, flow rate, and phase feedback signals—guaranteeing strict temporal alignment between all input control and output feedback signals. Acquired analog signals are converted via A/D conversion and processed in real time by an embedded processor. Using built-in algorithms, key performance indicators of the digital pump, such as outlet pressure, displacement, and efficiency, are calculated and monitored in real time, enabling dynamic evaluation of flow distribution effectiveness and system performance under varying electromagnetic phase angles.

### 4.2. Analysis of Key Actuator Parameter Effects

The stepless flow regulation system operates on a time-division control principle, achieving dynamic balance control of preset flow rates through precise phase-delayed closure adjustment of the suction valve. This enables accurate periodic matching between quantitative return flow and compression. Under varying load conditions, the system must fulfill multi-objective cooperative control requirements:

(1) The actuator must execute three-stage precision motion control of the suction valve spool (maintained opening–rapid closing–specified motion trajectory);
(2) Dual constraints of adequate suction and return flow control accuracy must be satisfied;
(3) Valve service life must be simultaneously considered.

With system energy efficiency guaranteed, a multi-parameter coupling analysis methodology is adopted: The first phase focuses on investigating the influence patterns of structural parameters (cylinder diameter, spring stiffness, unloader equivalent mass, etc.) and geometric parameters (control chamber volume, sealing length, etc.) on actuator displacement characteristics. The second phase is devoted to thoroughly analyzing the impact mechanisms of dynamic parameters (opening force, actuation phase, opening displacement, etc.) on both the dynamic characteristics of the suction valve (opening/closing response, flow pulsation, etc.) and the cyclic performance of the pump. Finally, based on the multi-objective optimization theory, with the aim of achieving indicators such as digital flow distribution, system regulation performance, and optimization of key parameters of the actuator, the global collaborative optimization of system parameters was completed.

**Figure 11:**
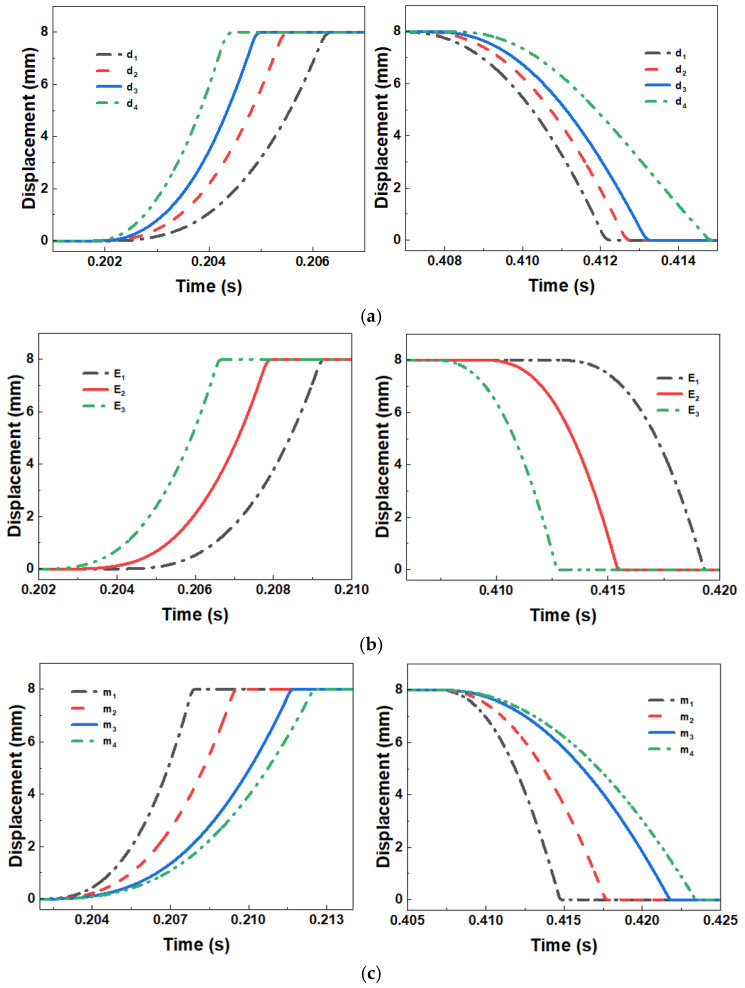

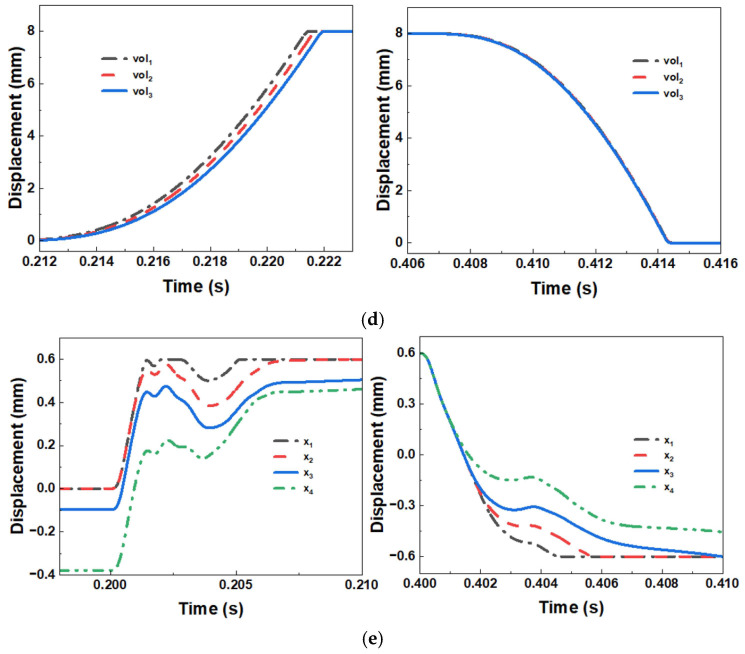
Comparison of the influence of different actuator parameters: (**a**) Cylinder diameter, (**b**) Spring stiffness, (**c**) Equivalent mass, (**d**) Control chamber volume, (**e**) Seal length.

Figure 11a presents the load–displacement characteristic curves under different hydraulic cylinder plunger diameters. Comparative analysis yields the following key conclusions:

When the plunger diameter increases from 10 mm to 14 mm, the system demonstrates marked dynamic performance improvements:

(1) The load opening delay time is decreased from 17 ms to 14.7 ms, a reduction of 13.5%;
(2) The average opening velocity increases by 22%.

This enhancement originates from the quadratic growth of the hydraulic effective area (a 96% increase in area), which substantially raises the hydraulic driving force under identical working pressure (a theoretical increase of 96%). Experimental data confirm that enlarging the plunger diameter not only significantly improves the initial acceleration of the actuator (by 40%) but also enhances the system’s capacity to drive inertial loads (a 95% rise in peak driving force). Accordingly, within the permissible range of system stiffness, the plunger diameter can be appropriately increased (recommended range: 12–14 mm) to effectively optimize the dynamic response characteristics of the actuator while ensuring structural reliability.

As shown in Figure 11b, reduction in equivalent load mass leads to significant improvement in the dynamic response characteristics of the actuator, specifically manifested as:

(1) Enhanced opening/closing response speed—when equivalent load mass decreases from 5 kg to 1 kg, the opening delay time is reduced by 18.8% (16 ms → 13 ms) and the closing delay time by 35.6% (26.1 ms → 16.8 ms);
(2) An increase in motion acceleration of approximately 40%.

In the optimization design of unloaders, advanced technologies such as topology-optimized structures and high-strength composite materials can be employed to maintain equivalent load mass within 1–3 kg while ensuring system rigidity and strength, thereby achieving optimal dynamic response performance (response time ≤ 15 ms).

As shown in Figure 11c, the influence pattern of spring stiffness on the actuator exhibits notable asymmetry:

(1) The influence coefficient reaches 0.85 during the closing phase, substantially higher than the 0.35 during opening;
(2) With every 100 N/mm increase in spring stiffness, the closing speed improves by approximately 22%, while the opening speed changes by only about 5%.

Theoretical analysis and experimental data demonstrate that when spring stiffness is maintained within 400–600 N/mm, the system achieves both fast closing characteristics (closing time < 20 ms) and avoids impact vibration caused by excessive stiffness (peak acceleration < 50 m/s^2^).

Figure 11d presents the load dynamic response characteristic curves under different hydraulic cylinder control chamber volumes. Systematic analysis yields the following key conclusions:

(1) A strong correlation is observed between control chamber volume and the dynamic performance of the actuator—when volume is reduced from 50 cm^3^ to 20 cm^3^, the opening delay time decreases by 32% (15 ms → 10.2 ms) and the closing delay time by 28% (22 ms → 15.8 ms);
(2) The stiffness effect induced by compressibility of hydraulic oil significantly impacts system stability, where each 100 cm^3^ of control chamber volume at a working pressure of 35 MPa generates approximately 0.5 mm of retraction of the unloader.

Based on fluid–structure interaction simulations and experimental validation, the control chamber volume should be optimized to the range of 15–25 cm^3^. For engineering implementation, advanced manufacturing techniques such as monobloc cylinder design and flow channel topology optimization can be employed to minimize control chamber volume while maintaining essential oil flow capacity.

When a negative overlap design is employed, stable operation of the spool is achieved after crossing the neutral position, albeit accompanied by significant pressure surge phenomena. Experimental data (Figure 11e) demonstrate that optimization of the spool sealing length yields the following improvements:

(1) A 30–40% reduction in peak pressure surge (specific value depends on working pressure level);
(2) A decrease in spool stabilization time of approximately 25%.

For structural optimization of the secondary hydraulic directional valve, optimal comprehensive performance is achieved when the sealing length is maintained within 0.5–1.2 mm. It should be particularly noted that reducing the sealing length introduces two engineering challenges:

(1) Stricter requirements for matching clearance tolerances;
(2) Enhanced specifications for spool surface finish.

**Figure 12:**
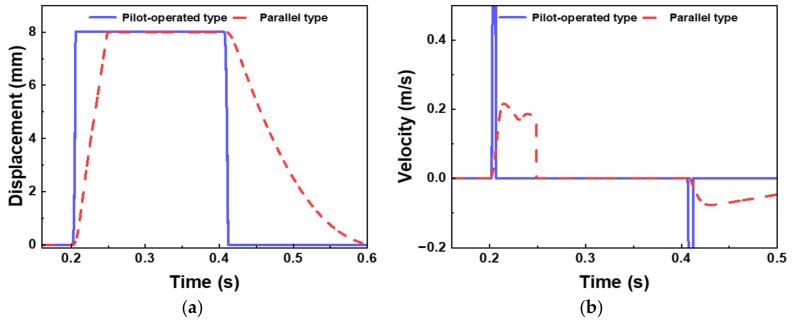
Comparison of actuator input pulse parameters: (**a**) Displacement, (**b**) Velocity.

Therefore, in practical engineering design, an optimal balance among response speed, leakage control, and manufacturing cost should be achieved through comprehensive fluid dynamics simulation and process feasibility analysis. Systematic analysis of the simulation waveforms yields the following key conclusions:

(1) Regarding command response characteristics, the pressure in the actuator control chamber demonstrates excellent dynamic response capability, with delay times for pressure build-up and release of only 12.5 ms (opening) and 15.3 ms (closing) after receiving actuation commands;
(2) Concerning motion transmission characteristics, as shown in Figure 12, a significant motion transmission delay is observed between the load displacement signal and the command signal, measuring 28.4 ms for the opening stroke and 34.7 ms for the closing stroke.

Experimental data confirm that optimizing the control chamber volume (recommended ≤25 cm^3^) and increasing system stiffness (recommended ≥500 N/μm) can effectively reduce motion transmission delays by approximately 35–40%, thereby significantly enhancing the overall system response performance.

### 4.3. Dynamic Characteristics of Different Flow Regulation Methods

**Figure 13:**
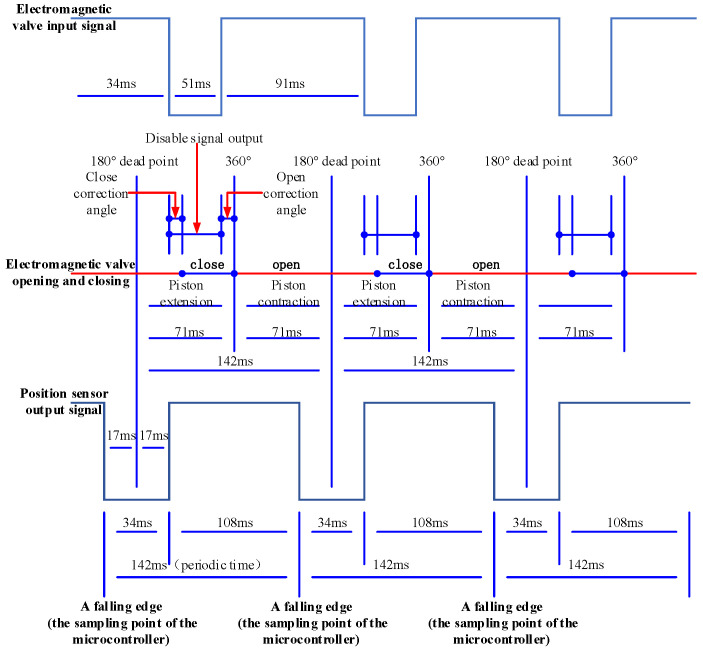
Timing diagram.

Based on the operational principles of the flow control system, the theoretical model governing partial discharge reflux conditions, and the analysis of cyclic regulation modes under varying fluid demands, the temporal relationships among four key parameters—piston rod extension/retraction, position sensor output, solenoid valve input signal, and its switching action—at specific delay phases can be established, as illustrated in Figure 13. The central red line in the diagram denotes the open/closed state of the solenoid valve. As a normally open valve is employed here, it remains in the open state initially. The position sensor detects the time interval *t* (where *t* = 17 ms in the figure) between the falling edge and the 180° top dead center (TDC). After a subsequent interval *t***,** an activation signal is dispatched to the solenoid valve. Following a closing correction delay Δ*t*_1_**,** the valve closes. When system operation is commanded, the solenoid valve is de-energized, and after an opening correction delayΔ*t*_2_**,** it reopens precisely upon reaching the 360° extreme position.

The interval from 180° TDC to the 360° position constitutes half a cycle (T/2 = 71 ms), while the period between consecutive falling edges defines one full cycle (T = 142 ms). Successive cycles follow this same sequence. The duration *t* governs the magnitude of system displacement. Denoting the sustained opening and closing durations of the solenoid valve as *t_0_*, and *t_c_*, respectively, the following expressions apply:

(7) $$t_{o}=T/2+t+\Delta t_{1}$$

(8) $$t_{c}=T/2-t-\Delta t_{2}$$

To enhance control precision, the following timing constraints must be satisfied: the maximum actuator extension time shall not exceed the instant at which the suction valve spool initiates closure from its fully open position. Moreover, the spool dwell time at maximum opening (i.e., phase delay) should be dynamically adapted in response to actual fluid demand and control objectives. To ensure motion reliability, the retraction speed of the actuator must remain below the natural return speed of the valve spool in the absence of external actuation, thereby preventing mechanical interference.

**Figure 14:**
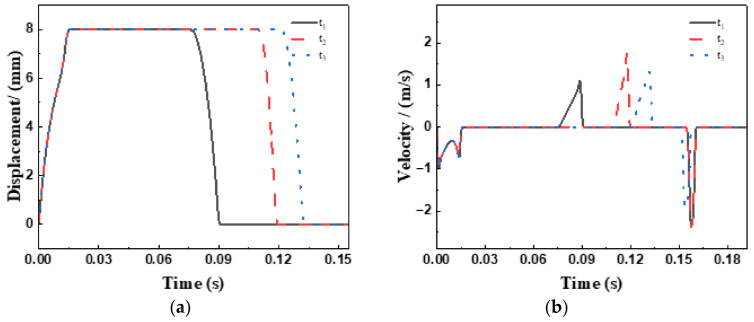
Comparison of actuator parameters under different time-delay states: (**a**) Displacement, (**b**) Velocity.

As evidenced by simulation results in Figure 14, precise regulation of reflux timing during the discharge stroke is achievable by modulating the actuator’s phase delay at the maximum opening position, thus fulfilling the technical requirements for active suction valve control. The underlying regulatory mechanism operates as follows: by adjusting the delayed closing time of the spool, a portion of the emulsion previously drawn into the plunger chamber (approximately 30–70% by volume) is permitted to return to the fluid reservoir via the suction valve. This effectively attenuates the flow rate delivered to downstream systems through the discharge valve, with the regulation amplitude reaching up to 60%.

**Figure 15:**
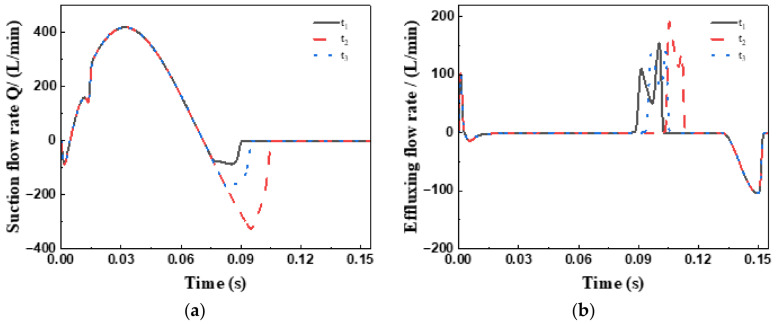
Comparison of flow rate curves: (**a**) Suction flow rate, (**b**) Discharge flow rate.

**Figure 16:**
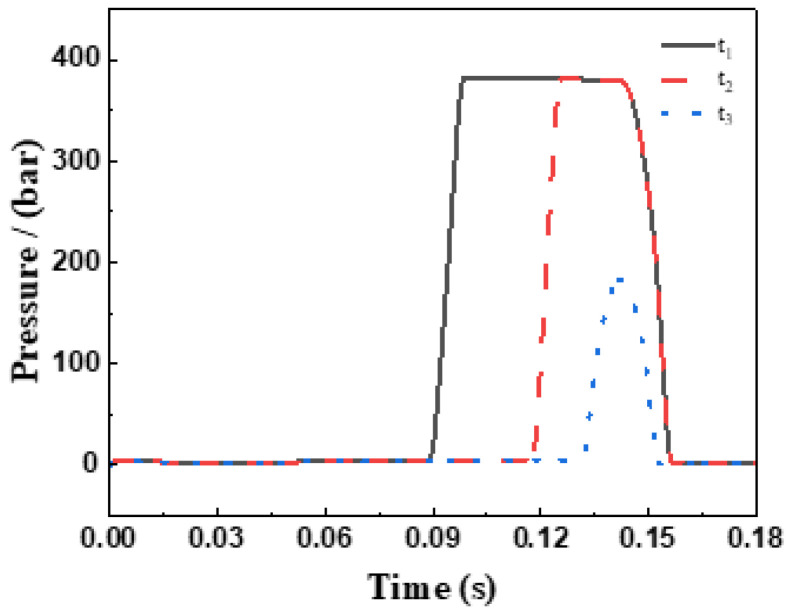
Pressure curve of the plunger chamber.

The characteristic flow profiles presented in Figure 15 and Figure 16 reveal the following patterns:

(1) Positive flow segments ( ) correspond to the hydraulic medium entering the plunger chamber through the valve;
(2) Negative flow segments ( ) indicate medium returning from the plunger chamber;
(3) The net flow exchange per cycle is quantitatively captured by the envelope area of these curves.

Both the net inflow to the plunger chamber ( ) and the single-plunger discharge capacity ( ) exhibit monotonically decreasing trends as the actuator return time increases.

To further investigate and compare the effectiveness of the digital flow distribution method, and to meet the requirements of intelligent mining for precise flow control, this paper conducts a comparative study on the flow regulation methods of digital unloading versus traditional unloading valves in emulsion pumps under various working conditions.

**Figure 17:**
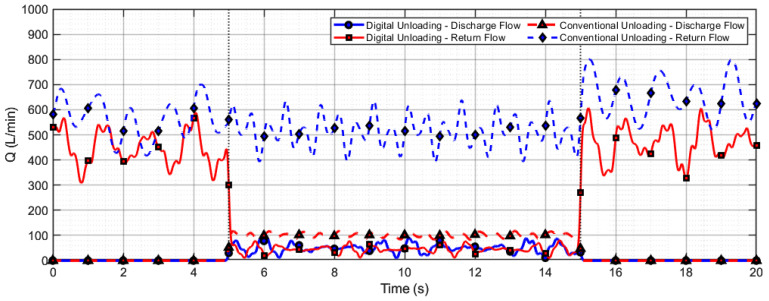
Flow comparison between digital and conventional unloading during lowering operation.

The flow performance of digital unloading and conventional valve unloading methods during the lowering operation is compared in Figure 17. Under the digital unloading strategy, the system rapidly reduces the supply flow from 450 L/min to the required 80 L/min for a single support lowering operation, and quickly restores high flow supply after the action is completed, demonstrating its capability for fast and precise on-demand flow control. In contrast, with the conventional valve unloading method, the average discharge flow is 500 L/min, while the return flow through the unloading valve reaches as high as 400 L/min, indicating that most of the flow is bypassed back to the tank. A comparison of the flow characteristics between the two methods reveals that the digital unloading approach effectively avoids frequent opening and closing of the unloading valve, thereby reducing energy loss and suppressing flow pulsation.

**Figure 18:**
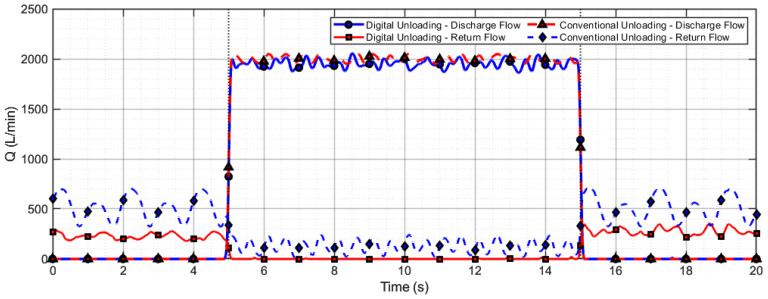
Flow comparison between digital and conventional unloading during raising operation.

A comparison of the flow performance between digital unloading and conventional valve unloading methods during the raising operation is shown in Figure 18. To meet the high flow demand in this process, a system consisting of four pumps in parallel was used for fluid supply. Under the digital unloading strategy, the average total supply flow of the pumping station was 1900 L/min, with no return flow (0 L/min) observed at the unloading valve of each pump. In contrast, under the conventional unloading strategy, the total supply flow reached 2000 L/min, resulting in a return flow of approximately 500 L/min per pump through the unloading valves. These results indicate that the digital unloading method can completely avoid return flow loss during the raising operation, even in a multi-pump parallel configuration.

**Figure 19:**
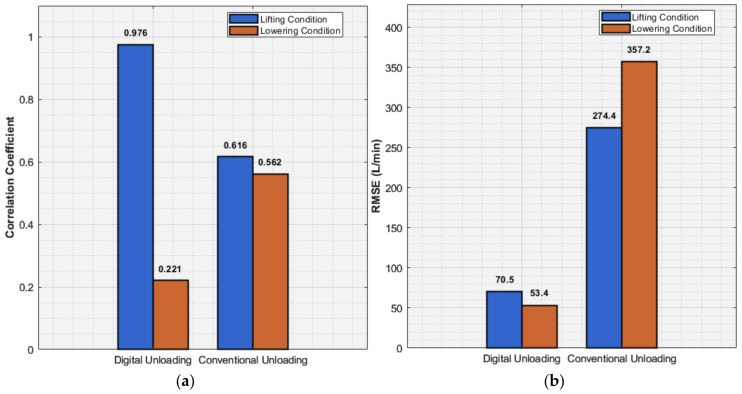
Performance comparison under extreme conditions: (**a**) correlation coefficient; (**b**) RMSE.

Figure 19 systematically evaluates the performance of digital unloading and traditional unloading under extreme operating conditions using two key metrics: the correlation coefficient and the root mean square error (RMSE). As shown in Figure 19a, in the comparison of correlation coefficients reflecting control consistency, digital unloading achieves an excellent level of 0.976 under lifting conditions, significantly outperforming the 0.616 of traditional unloading. This indicates that the flow output of digital unloading aligns closely with the target value, highlighting its superior linear control characteristics. The RMSE results in Figure 19b further confirm its precision advantage: under lifting conditions, the error of digital unloading is only 70.5 L/min, while that of traditional unloading reaches 274.4 L/min, revealing a substantial gap in control performance between the two methods.

The root cause of this performance difference lies in their distinct core regulation mechanisms. The digital unloading method achieves demand-based flow distribution at the source through precise phase control, maintaining near-ideal control linearity (R^2^ > 0.97) even under extreme conditions. In contrast, the traditional unloading valve relies on the throttling and overflow principle. Its performance deteriorates sharply after exceeding 80% load, with a significantly reduced regulation linearity (R^2^ ≈ 0.61). Moreover, it generates approximately 500 L/min of return flow loss under lifting conditions. This inherent throttling mechanism not only leads to energy waste but also directly results in substantial flow errors and pressure pulsations, which are the primary reasons for its high RMSE values.

In summary, the digital unloading method demonstrates comprehensive and robust performance advantages. Although its dynamic process is more complex during lowering conditions, resulting in a slightly lower correlation coefficient (0.221) compared to traditional unloading (0.562), its RMSE (53.4 L/min) remains significantly lower than that of traditional unloading (357.2 L/min). This confirms its higher practical control accuracy under complex operating conditions. Combined with the smaller flow fluctuation observed in repeatability tests (±3.5 L/min vs. ±12.8 L/min), it can be concluded that by avoiding frequent valve switching and return flow losses, digital unloading achieves near-ideal control performance under lifting conditions while maintaining superior accuracy and stability under lowering conditions. Its outstanding overall performance makes it particularly suitable for intelligent mining equipment operating under high-pressure and large-flow conditions with stringent control quality requirements.

## 5. Conclusions

This study successfully addresses the limitations of traditional emulsion pump stations by developing and validating an intelligent digital flow control system. The core achievements are summarized as follows:

(1) Verified digital flow regulation: A multi-physics field collaborative simulation model was established, which can accurately capture the dynamic interactions among the pilot electromagnetic valve, hydraulic pulsation, and actuator movement. The experimental results show that by precisely controlling the phase angle of the pilot valve closure to achieve the delay of the actuator, the pump output flow can be regulated. This system achieved a flow control range of up to 83% under load conditions, significantly better than the 57% range observed under no-load conditions. This indicates that it has a wider control range in dealing with actual load fluctuations.
(2) Synergistic Multi-Mode Compound Control Strategy: By leveraging Digital Displacement Pump (DDP) principles, a compound strategy integrating single-cycle, dual-cycle, and multi-cycle operations with adjustable duty cycles was implemented. This approach expands the pump’s effective flow range from 20% to 100% of its rated capacity. More importantly, the synergistic optimization of solenoid valve timing reduces component actuation frequency and minimizes overflow losses, leading to a substantial 15–20% improvement in overall system energy efficiency compared to conventional variable-frequency drives.
(3) Mechanism-driven Parameter Optimization: A systematic parameter sensitivity analysis revealed that a 35–40% reduction in spool retraction speed, coupled with the coordinated optimization of return spring stiffness (400–600 N/mm) and return oil pressure (0.5–1.2 MPa), effectively suppresses pressure surges and flow pulsations. This insight explains the origin of residual flow under no-load conditions and provides clear guidance for achieving more stable and efficient system designs.
(4) Superior Performance of the Multi-Sensor Digital Unloading Strategy: The proposed digital unloading strategy overcomes the slow response and hysteresis of traditional methods. Based on a dynamic mapping model, it achieves a control correlation coefficient of 0.976 under lifting conditions, far surpassing the 0.616 of the conventional method, while reducing the flow root mean square error to 70.5 L/min (vs. 274.4 L/min). This provides a key technical solution for intelligent, adaptive flow control in hydraulic support systems.

### 5.1. Innovations

An intelligent flow control method based on the digital flow distribution principle is proposed, which enables active perception and matching of support demands. This fundamentally transforms the traditional passive response regulation mode of fluid supply systems, achieving precise and smooth flow delivery while suppressing system fluctuations and shocks at the source. A structurally compact, electro-hydraulically separated prototype for stepless flow regulation is developed, providing a reliable hardware implementation platform for the aforementioned control method and supporting the system’s miniaturization and integrated design. An optimized combined regulation strategy integrating adjustable duty cycles across single, dual, and multiple cycles is introduced, significantly enhancing the system’s dynamic response capability, control flexibility, and overall reliability under complex working conditions. These synergistic innovations at the methodological, structural, and strategic levels collectively provide a systematic solution for achieving precise, efficient, and reliable control of the fluid supply system for hydraulic supports.

### 5.2. Limitations and Future Work

The limitations of this study are primarily focused on three aspects. First, the scope of control objects requires expansion. The current research concentrates on flow matching for the core actions of the support, lacking sufficient verification concerning the complex collaborative fluid supply demands and dynamic superposition effects of full-process operations, which include auxiliary actions. Second, the long-term reliability of the actuator remains unclear. Its performance stability and lifespan degradation patterns under high-speed and high-frequency operating conditions necessitate verification through more stringent durability tests. Third, the intelligent adaptive capability of the control strategy needs enhancement. The existing strategy struggles to adjust and optimize objectives online based on real-time fluctuations in working conditions. Addressing these limitations, future research will focus on three main directions: constructing a refined full-process action spectrum, developing adaptive control algorithms that integrate real-time perception and online learning, and enhancing system durability and intelligent decision-making levels through hardware-in-the-loop and industrial field tests. These efforts aim to advance fluid supply systems toward higher reliability, responsiveness, and adaptability.

## Figures and Tables

**Table 1:** Spring parameters.

Items	Reset Spring	Valve Plate Spring
Spring free height/mm	45.4	55.9
Spring stiffness/(N/mm)	4	5
Pitch/mm	4.5	7.5
Diameter of the steel wire/mm	2	3
Effective turns	8.75	6.25
Total number of laps	10.75	8.25

**Table 2:** Geometric and operational parameters of the BRW630/37.5 pump.

Items	Value
Crankshaft Speed/(r/min)	422
Crankshaft Radius/mm	55
Connecting Rod Length/mm	335
Plunger Diameter/mm	60
Plunger Stroke/mm	110
Valve Spool Mass/g	365/270
Valve Spool Stroke/mm	8/6.7
Valve Spring Stiffness/(N/mm)	1.844/2.1
Valve Spool Diameter/mm	91/60

**Table 3:** Key parameters of the sensor.

Items	Model	Range	Precision	Output
Pressure Sensor	CYG1508 (Guizhou Honglin Aviation Power Control Technology Co., Ltd. of China Aviation Industry Corporation, Guiyang, China)	0~50 Mpa	0.1%FS	0~5 V
Hall Sensor	CZ400-A0100-B02-C00 (Guizhou Honglin Aviation Power Control Technology Co., Ltd. of China Aviation Industry Corporation, Guiyang, China)	0~20 kHz	0.1%FS	0~5 V
Flow Sensor	LWGY-15 (Guizhou Honglin Aviation Power Control Technology Co., Ltd. of China Aviation Industry Corporation, Guiyang, China)	17~170 L/min	0.5%	0~5 V

## Data Availability

The original contributions presented in this study are included in the article. Further inquiries can be directed to the corresponding author.

## Nomenclature

$x_{p}$	Plunger displacement (m)	$Q_{s}/Q_{d}$	Instantaneous flow rates through the suction valve and discharge valve (m^3^·s^−1^)
$v_{p}$	Velocity of the plunger (m·s^−1^)	$Q_{p}$	Volume change rate caused by the plunger movement (m^3^·s^−1^)
$F_{smu}$	Spring force of the unloader (N)	$Q_{0}$	Flow rate at the valve orifice (m^3^·s^−1^)
$F_{L}$	Ejection force from the hydraulic cylinder (N)	$V_{vol}$	Instantaneous volume (m^3^)
f	Total friction force (N)	$V_{d}$	Dead volume of the plunger chamber (m^3^)
$P_{h}$	Hydraulic driving force (N)	$t_{i}$	Time of unloader ejection (*i* = 1, 2, 3) (s)
$P_{1}/P_{2}$	Pressure in the left/right chamber of the valve spool (Pa)	$C_{d}$	Flow discharge coefficient of the valve orifice
$P_{vol}$	Instantaneous pressure in the plunger chamber (Pa)	TDC/BDC	Top Dead Center/Bottom Dead Center
$x/x_{mu}$	Displacement of the valve spool/unloader (m)	Greek	
$M_{mu}$	Mass of the unloader (kg)	ρ	Fluid density
E	Bulk modulus of the liquid in the chamber (Pa)	θ	crank angle
$A_{v}/A_{p}$	Cross-sectional area of the plunger (m^2^)		
